# Concomitant genetic alterations having greater impact on the clinical benefit of EGFR‐TKIs in *EGFR*‐mutant advanced NSCLC than *BIM* deletion polymorphism

**DOI:** 10.1002/ctm2.12

**Published:** 2020-05-19

**Authors:** Si‐Yang Liu, Jia‐Ying Zhou, Wen‐Feng Li, Hao Sun, Yi‐Chen Zhang, Hong‐Hong Yan, Zhi‐Hong Chen, Chun‐Xiang Chen, Jun‐Yi Ye, Jin‐Ji Yang, Qing Zhou, Xu‐Chao Zhang, Yi‐Long Wu

**Affiliations:** ^1^ Guangdong Lung Cancer Institute, Guangdong Provincial People's Hospital, Guangdong Academy of Medical Sciences, School of Medicine South China University of Technology Guangzhou 510080 China; ^2^ Burning Rock Biotech Guangzhou 510000 China

**Keywords:** *BIM* deletion polymorphism, concomitant genetic alterations, EGFR‐TKIs, next‐generation sequencing, NSCLC

## Abstract

**Background:**

In previous studies, the predictive role of *BIM* deletion polymorphism with respect to responses to epidermal growth factor receptor tyrosine kinase inhibitors (EGFR‐TKIs) has been controversial. The potential reasons for these inconsistent findings were unknown.

**Methods:**

Data from CTONG0901 clinical trial and medical records of Guangdong Lung Cancer Institute (GLCI) were retrospectively pooled. A total of 194 and 141 *EGFR*‐mutant non‐small cell lung cancer (NSCLC) patients treated with first‐ and second‐generation EGFR‐TKIs were examined in the CTONG0901 and GLCI cohorts, respectively. Sixty‐eight patients were treated with third‐generation EGFR‐TKIs in the GLCI cohort. The *BIM* gene status was examined by next‐generation sequencing.

**Results:**

The frequency of *BIM* deletion polymorphism was 11.3% and 17.0% in CTONG0901 and GLCI cohorts, respectively. For first‐ and second‐generation EGFR‐TKIs in CTONG0901 cohort, objective response (ORR) was 54.5% in *BIM* deletion group versus 56.4% in wild‐type *BIM* group (*P* = .87); disease control rate (DCR) was 90.9% versus 88.4% (*P* = 1.00); progression‐free survival (PFS) was 10.5 versus 11.2 months (*P* = .59); and overall survival (OS) was 20.5 versus 20.5 months (*P* = .73). In GLCI cohort, ORR was 54.2% versus 60.7% (*P* = .55); DCR was 91.7% versus 96.6% (*P* = .27); PFS was 10.1 versus 11.6 months (*P* = .63); and OS was 58.5 versus 45.0 months (*P* = .93). For third‐generation EGFR‐TKIs, ORR was 18.2% versus 63.2% (*P* = .02); DCR was 81.8% versus 96.5%, (*P* = .12); PFS was 5.8 versus 9.0 months (*P* = .13); and OS was 30.0 versus 24.8 months (*P* = .85). Cox regression analysis showed that concomitant genetic alterations could adversely affect the response to EGFR‐TKIs, but not *BIM* deletion.

**Conclusions:**

The presence of *BIM* deletion showed no relation to an impaired response to first‐, second‐, and third‐generation EGFR‐TKIs in NSCLC patients. The factors influencing the response of EGFR‐TKIs were concomitant genetic alterations, but not *BIM* deletion.

**Abbreviations**
CML: chronic myeloid leukemia
EGFR‐TKIs: epidermal growth factor receptor tyrosine kinase inhibitors
NGS: next‐generation sequencing
GLCI: Guangdong Lung Cancer Institute
NSCLC: nonsmall cell lung cancer
PFS: progression‐free survival
OS: overall survival
ORR: objective response rate
HR: hazard ratio
CI: confidence interval

## 1 BACKGROUND

Previous reports have indicated that the incidence of epidermal growth factor receptor (*EGFR*) mutations is high in Asian populations, reaching 30% in non‐small cell lung cancer (NSCLC) patients and 50% in those with adenocarcinoma.^1^ Patients with *EGFR* mutations can be treated with first‐, second‐, and third‐generation epidermal growth factor receptor tyrosine kinase inhibitors (EGFR‐TKIs), in a first‐line setting and beyond.^2^, ^3^, ^4^, ^5^, ^6^ *BIM* is a pro‐apoptotic member of the B‐cell lymphoma‐2 family and plays a role in regulating apoptosis during tumor formation.^7^ Activated *BIM* exerts a pro‐apoptotic action through various pathways by translocation to the mitochondrial membrane.^8^ *BIM* contains only one BH3 domain, which is essential for the pro‐apoptotic activity of each *BIM* subtype.^9^ EGFR‐TKIs upregulate *BIM* expression to induce apoptosis of lung cancer cells with *EGFR* mutations.^10^, ^11^ Therefore, decreased *BIM* expression in malignant tumor inhibits tumor cell apoptosis and promotes tumor development. *BIM* deletion polymorphisms are common in Asian populations, with an incidence of 12‐16% in lung cancer patients with *EGFR* mutations.^12^, ^13^ Previous studies have shown that patients with *EGFR* mutations and *BIM* deletion polymorphisms are less responsive to EGFR‐TKI therapy.^14^, ^15^, ^16^ In contrast, other studies showed that *BIM* deletion polymorphism had no effect on the progression‐free survival (PFS) or overall survival (OS) of patients treated with EGFR‐TKI therapy.^13^, ^17^

The role of *BIM* in the induction of apoptosis of lung cancer cells, and its involvement in the primary resistance to EGFR‐TKIs of lung cancer patients, has attracted attention. The available data are inconsistent regarding the predictive role of *BIM* deletion polymorphisms. With the advent of next‐generation sequencing (NGS), concomitant genetic alterations are increasingly detected. Comprehensive genetic analysis is needed to understand the inconsistent outcomes and to identify which genetic variants have an impact on the responsiveness to EGFR‐TKIs. Here, we used NGS to examine the predictive and prognostic roles of *BIM* deletion polymorphisms with respect to the response to first‐, second‐, and third‐generation EGFR‐TKIs in NSCLC patients from two independent cohorts.

## 2 METHODS

### 2.1 Patients

A total of 256 *EGFR*‐mutant patients diagnosed with advanced‐stage NSCLC were enrolled in the CTONG0901 clinical trial. The first‐generation EGFR‐TKIs, erlotinib or gefitinib, were given to patients as any‐line treatment. Of the patients, 194 had sufficient tumor tissue at baseline for analysis by NGS using a panel of 168 genes; 22 of these patients had *BIM* deletion polymorphism and 172 had the wild‐type *BIM*.

From January 2016 to July 2018, the clinical data of 141 NSCLC patients with *EGFR* mutations from Guangdong Lung Cancer Institute (GLCI) were retrospectively pooled. Patients were treated with first‐ or second‐generation EGFR‐TKIs as any‐line treatment. The *EGFR* mutation status was identified by an amplification refractory mutation system or NGS. *BIM* deletion polymorphism represents a germline mutation, so the status of *BIM* was assessed by NGS at least once during the treatment process. Twenty‐four patients had *BIM* deletion polymorphism and 117 had wild‐type *BIM*. In later lines of treatment, 68 patients were treated with third‐generation EGFR‐TKIs (11 patients in *BIM* deletion group and 57 in wild‐type *BIM* group).

### 2.2 NGS at baseline

A total of 194 patients in the CTONG0901 cohort (22 patients: *BIM* deletion; 172 patients: wild‐type *BIM*) and 29 in the GLCI cohort (seven patients: *BIM* deletion; 22 patients: wild‐type *BIM*) had NGS results at the baseline of first‐ and second‐generation EGFR‐TKIs. Thus, 223 patients were included in the final analysis of concomitant mutations and survival (29 patients: *BIM* deletion; 194 patients: wild‐type *BIM*). Fresh frozen or formalin fixed paraffin‐embedded tissues from 217 patients, and peripheral plasma or cerebrospinal fluid specimens from six patients, were used for NGS testing. A total of 214 samples were analyzed with a panel of 168 genes; nine samples were analyzed with panels of 295 or 520 genes. The NGS results of 168 overlapping genes were included in the final analysis.

### 2.3 Analysis

The clinical and pathological characteristics of patients with and without *BIM* deletion polymorphism were analyzed with SPSS version 22.0 and GraphPad Prism version 7.00. The chi‐square test was used to analyze categorical data. PFS was measured from the date of EGFR‐TKI treatment to the date of disease progression or last follow‐up. OS was measured from the date of EGFR‐TKI treatment to the date of death or last follow‐up, with a cutoff date of June 2019. Kaplan‐Meier survival curves were generated to estimate PFS and OS in the *BIM* deletion and wild‐type *BIM* groups. Univariate analysis and multivariate Cox regression analysis were performed to determine whether the alterations in genetic background had an impact on PFS or OS in patients treated with EGFR‐TKIs, in addition to *BIM* deletion polymorphism. The predictive and prognostic factors investigated included 167 genetic alterations, *BIM* deletion, PFS, and OS.

## 3 RESULTS

### 3.1 Clinical and pathological characteristics of *BIM* deletion polymorphism

The incidence rates of *BIM* deletion polymorphism in *EGFR*‐mutant patients with advanced‐stage NSCLC were 11.3% (22/194) and 17.0% (24/141) in the CTONG0901 and GLCI cohorts, respectively. Homozygous *BIM* deletion only occurred in 4.3% of patients (2/46).

The distribution of clinical and pathological characteristics in patients with and without *BIM* deletion in the CTONG0901 and GLCI cohorts is summarized in Table 1. There were no significant differences in age, sex, smoking history, Eastern Cooperative Oncology Group performance status score, pathology, or clinical stage between the *BIM* deletion and wild‐type *BIM* groups.

**TABLE 1:** The clinical and pathological characteristics of *EGFR*‐mutant patients with and without *BIM* deletion polymorphism in the CTONG0901 and GLCI cohorts

	CTONG0901 cohort			GLCI cohort		
	*BIM* deletion (n = 22)	Wild‐type *BIM* (n = 172)	*P*‐value	*BIM* deletion (n = 24)	Wild‐type *BIM* (n = 117)	*P*‐value
Age in years, mean ± SD	59.1± 11.2	58.7± 11.2		57.6 ±10.4	59.1± 11.2	
Age in years, n (%)						
<65	17 (77%)	121 (70%)	.50	20 (83%)	99 (85%)	1.00
≥65	5 (23%)	51 (30%)		4 (17%)	18 (15%)	
Sex, n (%)						
Male	12 (55%)	80 (47%)	.48	11 (46%)	50 (43%)	.78
Female	10 (45%)	92 (53%)		13 (54%)	67 (57%)	
Smoking histology, n (%)						
Never	17 (77%)	136 (79%)	.85	19 (79%)	96 (82%)	.74
Former/Current	5 (23%)	36 (21%)		5 (21%)	21 (18%)	
ECOG PS, n (%)						
0‐1	22 (100%)	169 (98%)	1.00	22 (92%)	113 (97%)	.27
≥2	0 (0%)	3 (2%)		2 (8%)	4 (3%)	
Pathology, n (%)						
Adenocarcinoma	22 (100%)	164 (95%)	.60	23 (96%)	113 (97%)	1.00
Squamous carcinoma	0 (0%)	3 (2%)		0 (0%)	0 (0%)	
Others	0 (0%)	5 (3%)		1 (4%)	4 (3%)	
Stage, n (%)						
IIIB	0 (0%)	5 (3%)	1.00	2 (8%)	4 (17%)	.27
IV	22 (100%)	167 (97%)		22 (92%)	113 (83%)	
*EGFR* mutations, n (%)						
19 deletion	12 (55%)	92 (54%)	.93	10 (42%)	66 (56%)	.19
21L858R mutation	9 (41%)	76 (44%)		14 (58%)	49 (42%)	
Others	1 (4%)	4 (2%)		0 (0%)	2 (2%)	
EGFR‐TKIs, n (%)						
First‐generation	22 (100%)	172 (100%)		24 (100%)	106 (91%)	.21
Second‐generation	–	–		0 (0%)	11 (9%)	
Line of first‐ and second‐generation EGFR‐TKIs, n (%)						
First line	19 (86%)	113 (66%)	0.09	20 (83%)	105 (90%)	.58
Second line and beyond	3 (14%)	59 (34%)		4 (17%)	12 (10%)	
Line of third‐generation EGFR‐TKIs, n (%)						
First line	–	–		1 (9%)	7 (12%)	.95
Second line	–	–		7 (64%)	34 (60%)	
Third line and beyond	–	–		3 (27%)	16 (28%)	

Abbreviations: ECOG PS, Eastern Cooperative Oncology Group performance status; *EGFR*, epidermal growth factor receptor; EGFR‐TKIs, epidermal growth factor receptor tyrosine kinase inhibitors.

### 3.2 Clinical efficacy of EGFR‐TKIs and *BIM* deletion polymorphism

**FIGURE 1:**
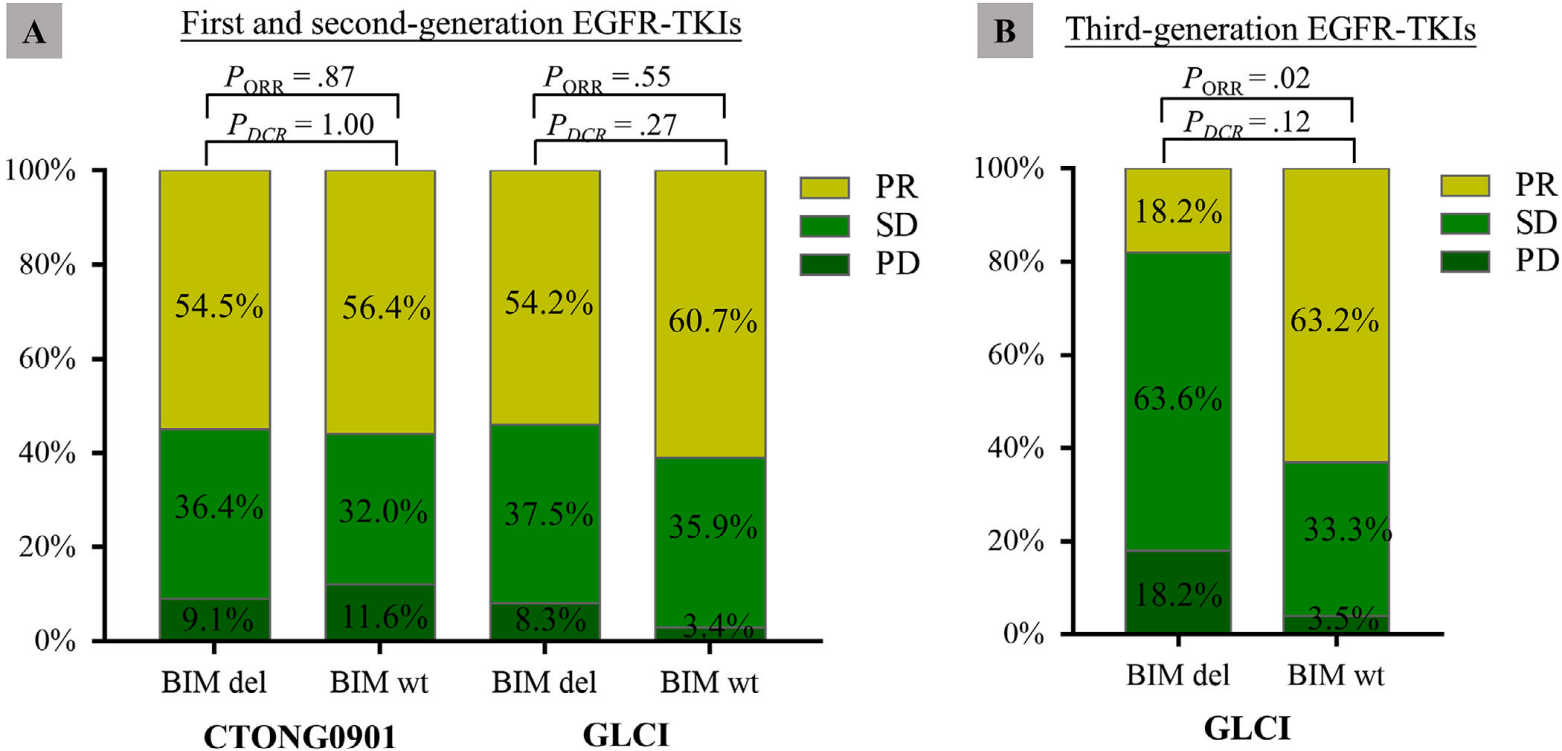
A, Comparable analysis of the clinical response of first‐ and second‐generation epidermal growth factor receptor tyrosine kinase inhibitors (EGFR‐TKIs) of patients with and without *BIM* deletion polymorphism. B, Comparable analysis of responsiveness to third‐generation EGFR‐TKIs of patients with and without *BIM* deletion polymorphism Abbreviations: *BIM* del, *BIM* deletion; *BIM* wt, wild‐type *BIM*.

**FIGURE 2:**
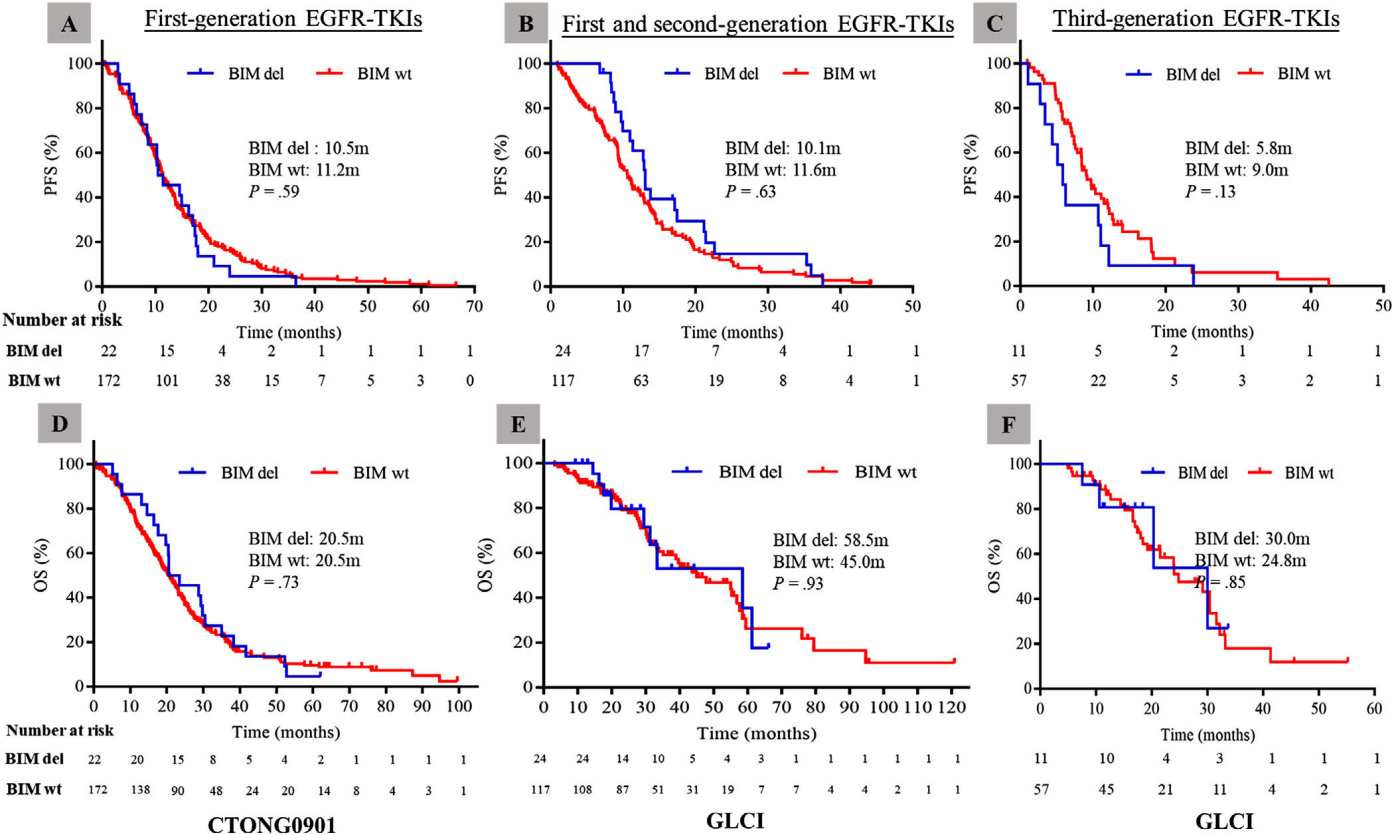
Survival analysis of the progression‐free survival (PFS) and overall survival (OS) of patients with and without *BIM* deletion polymorphism treated with first‐generation EGFR‐TKIs in the CTONG0901 cohort (A and D) and treated with first‐ and second‐generation EGFR‐TKIs in the GLCI cohort (B and E). Survival analysis of PFS and OS of patients with and without *BIM* deletion polymorphism treated with third‐generation EGFR‐TKIs in GLCI cohort (C and F) Abbreviations: *BIM* del, *BIM* deletion; *BIM* wt, wild‐type *BIM*.

The proportions of partial response, stable disease, and progressive disease were comparable between the *BIM* deletion and wild‐type *BIM* groups (Figure 1A). The objective response rate (ORR) was 54.5% versus 56.4% in patients with and without *BIM* deletion in the CTONG0901 cohort, respectively (*P* = .87), and 54.2% versus 60.7% in the GLCI cohort, respectively (*P* = .55). The disease control rate (DCR) was 90.9% versus 88.4% in patients with and without *BIM* deletion in the CTONG0901 cohort, respectively (*P* = 1.00), and 91.7% versus 96.6% in the GLCI cohort, respectively (*P* = .27). The median PFS and OS were not significantly different between patients with and without *BIM* deletion in the CTONG0901 cohort (PFS: 10.5 vs 11.2 months, respectively, *P* = .59; OS: 20.5 vs 20.5 months, respectively, *P* = .73) (Figures 2A and 2D). Similar results were obtained in the GLCI cohort (PFS: 10.1 vs 11.6 months, respectively, *P* = .63; OS: 58.5 vs 45.0 months, respectively, *P* = .93) (Figures 2B and 2E). Subgroup analysis was performed in patients with *EGFR* 19 deletions and 21L858R mutations. The median PFS and OS also showed no association with *BIM* status in either cohort (Figures S1 and S2). *BIM* deletion polymorphism was not associated with a poorer curative effect of EGFR‐TKIs in two independent cohorts.

Similar analyses were performed in patients treated with the third‐generation EGFR‐TKIs, osimertinib and avitinib. The ORR was lower in the *BIM* deletion group than in the wild‐type *BIM* group (18.2% vs 63.2%, respectively, *P* = .02). The DCR was 81.8% in the *BIM* deletion group and 96.5% in the wild‐type *BIM* group (*P* = .12) (Figure 1B). The median PFS and OS were comparable between the two groups (PFS: 5.8 vs 9.0 months, respectively, *P* = .13; OS: 30.0 vs 24.8 months, respectively, *P* = .85) (Figures 2C and 2F).

### 3.3 Relations of concomitant genetic alterations to EGFR‐TKI responsiveness

**FIGURE 3:**
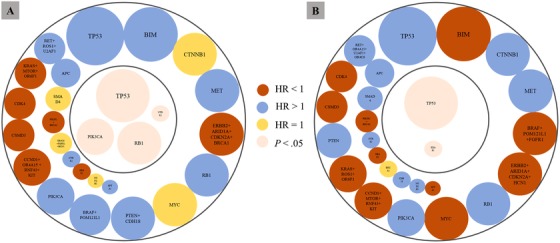
The result of cox regression analysis to identify which concomitant genetic variations were associated with impaired or enhanced curative effects of EGFR‐TKIs. *BIM* deletion and concomitant genetic variations detected in at least three patients were presented. A, Genetic factors influencing the PFS of patients treated with EGFR‐TKIs. B, Genetic factors influencing the OS of patients treated with EGFR‐TKIs. The outside circle represents the genes included in Cox regression analysis. The inside circle represents the results of the multivariate analysis; statistically significant *P*‐values are provided. The different colors and sizes of circles represent the hazard ratio and sample size of patients with corresponding genetic alterations, respectively

We examined the potential reasons for the inconsistent outcomes in previous studies regarding the relation between *BIM* deletion and the response to EGFR‐TKIs. Along with the results of NGS, all genetic alterations and *BIM* deletion polymorphisms were input into univariate and multivariate analyses to identify which concomitant genetic variations were associated with impaired or enhanced curative effects of EGFR‐TKIs. The heat map showed the *EGFR* mutation subtypes, *BIM* status, and concomitant genetic variations detected in at least three patients (Figure S3). The frequency of concomitant TP53 mutation was the highest, which was 58.6% (17/29) in *BIM* deletion group and 68.0% (132/194) in wild‐type *BIM* group. Further, we screened the genes identified by NGS to determine which had an influence on the PFS and OS of patients treated with EGFR‐TKIs. Cox multivariate regression analysis showed that four genetic alterations were associated with poorer PFS—*TP53* mutation (hazard ratio [HR] = 1.5; 95% confidence interval [CI], 1.10‐2.00; *P* = .01), *NTRK*1 mutation and amplification (HR = 4.7; 95% CI, 1.64‐13.40; *P* = .04), *RB1* mutation (HR = 1.7; 95% CI, 1.06‐2.8; *P* = .03), and *PIK3CA* mutation (HR = 1.7; 95% CI, 0.99‐3.00 *P* = .05) were the strongest independent predictors of shorter PFS in patients treated with EGFR‐TKIs (Figure 3A). In addition, two genetic alterations were shown to have an impact on OS in patients treated with EGFR‐TKIs—*TP53* mutation was the strongest independent predictor of a shorter OS (HR = 1.50; 95% CI, 1.10‐2.06; *P* = .01). *KEAP1* mutation and deletion was the strongest independent predictor of a longer OS (HR = 0.12; 95% CI, 0.02‐0.88; *P* = .04) (Figure 3B). Thus, neither analysis showed an effect of *BIM* deletion polymorphism on PFS and OS in NSCLC patients treated with EGFR‐TKIs.

## 4 DISCUSSION

In 2012, Ng reported that *BIM* deletion polymorphism mediated primary resistance to TKIs in cancers including chronic myeloid leukemia (CML) and *EGFR*‐mutant NSCLC; this; was confirmed by in vitro cell culture experiments and clinical data.^14^ Subsequently, two studies from Japan indicated that *BIM* deletion was associated with a reduced benefit of first‐ and third‐generations EGFR‐TKIs, based on the results of in vitro and in vivo experiments.^18^, ^19^ However, in 2014, this finding was challenged by Chinese researchers with the conclusion that the *BIM* deletion polymorphism cannot account for intrinsic TKI resistance of Chinese individuals with CML.^20^ Subsequent studies with clinical data in EGFR mutant NSCLC were inconsistent regarding the predictive role of *BIM* deletion polymorphism with respect to the responsiveness to first‐generation EGFR‐TKIs. Two studies from Korea^13^, ^17^ and one from China^21^ showed that *BIM* deletion polymorphism was not an independent predictor of a poor response to EGFR‐TKIs in *EGFR*‐mutant NSCLC patients, whereas other studies ^15^, ^16^, ^22^ and studies detecting *BIM* mRNA expression^23^, ^24^ reported the opposite results. However, *BIM* deletion polymorphism was not a prognostic biomarker for OS in most studies. In the present study, *BIM* deletion polymorphism had no influence on the PFS or OS of patients treated with first‐ and second‐generation EGFR‐TKIs in two independent cohorts. In general, almost half of the clinical studies did not replicate the results of cell line experiments regarding the effects of *BIM* deletion on the response to EGFR‐TKIs. It is inevitable for the occurrence of drug resistance after patients treated with first‐ or second‐generation of EGFR‐TKIs for 9‐12 months.^25^, ^26^ A total of 50% NSCLC patients carried *EGFR T790M* mutation and third‐generation EGFR‐TKIs would be the optimal choice for the next‐step treatment.^6^ To date, with the exception of one case report,^27^ there have been no reports based on clinical data regarding the impact of *BIM* status on the response to third‐generation EGFR‐TKIs. In our study, although patients with *BIM* deletion tended to have a poor response to osimertinib or avitinib, the DCR, PFS, and OS were comparable to those of patients without *BIM* deletion. The small sample size of patients treated with third‐generation EGFR‐TKIs likely leads to selection bias, which could explain the lower ORR in the *BIM* deletion groups.

This study verified our hypothesis that, in addition to *BIM* deletion polymorphism, the genetic background could also have an impact on the responsiveness to EGFR‐TKIs. The presence of *TP53*, *RB1*, and *PIK3CA* mutations reduced the response to TKIs. Here, we attempted to give an explanation of the inconsistent evidence regarding the role of *BIM* deletion in the responsiveness to EGFR‐TKIs and identify why some clinical reports could not replicate the results of in vitro and in vivo experiments. First, with the advent of NGS, the genetic background with hundred genes could be profiled and the results should be taken into account when analyzing the influential factors to clinical benefit of EGFR‐TKIs. The co‐mutation profile of NSCLC patients have been landscaped in previous study.^28^ It was reported that concomitant mutation was associated with reduced response and poor survival of EGFR‐TKIs.^29^ Our study found that patients with *TP53* or *RB1* mutation tended to show an impaired response to EGFR‐TKIs. However, the detection method of *BIM* status in published studies could not profile the genetic background.^13^, ^16^, ^17^ Second, the incidence of *BIM* deletion in *EGFR* mutant patients with NSCLC was around 15% in these previous reports^15^, ^21^ and in our study. The small sample sizes led to a large degree of heterogeneity in the *BIM* deletion group. When more patients combined with TP53 or RB1 mutation included in this group, the outcome would be that *BIM* deletion had a more deleterious response to EGFR‐TKIs than wild‐type *BIM*. Third, it is apparent that some studies with clinical data could not replicate the results of in vitro and in vivo experiments in other research. As we know, the cell lines were pure, carrying only one or two mutations. However, the situation in clinical practice is usually more complex, where the presence of certain confounding factors is inevitable.

This study had some limitations. First, although our study had the largest sample size of *EGFR*‐mutant patients with known *BIM* status reported to date, only 68 patients were included in the survival analysis of treatment with third‐generation of EGFR‐TKIs. Second, baseline NGS results were available for only 24 patients with *BIM* deletion, which may have been an insufficient sample size to examine the role of *BIM* status in Cox regression analysis. Third, the mechanism underlying the lack of effect of *BIM* deletion polymorphism on responsiveness to EGFR‐TKIs remains unclear.

## 5 CONCLUSION

Overall 11.3% and 17.0% *EGFR*‐mutant patients with advanced‐stage NSCLC in the CTONG0901 and GLCI cohorts had *BIM* deletion polymorphism, respectively, which had no relationship with any clinical or pathological factors. The presence of *BIM* deletion was not associated with impaired survival in patients treated with first‐, second‐, and third‐generation EGFR‐TKIs. Concomitant genetic alterations, but not *BIM* deletion polymorphism, had an influence on the clinical benefit of EGFR‐TKIs in patients with advanced NSCLC.

## CONFLICT OF INTEREST

The authors declare no conflict of interest.

## Supporting information

FIGURE S1: Survival analysis of the PFS and OS of patients with *EGFR* 19 deletion with and without *BIM* deletion polymorphism, in the CTONG0901 cohort (A, C) and in the GLCI cohort (B, D). *BIM* del: *BIM* deletion; *BIM* wt: wild‐type *BIM*.

FIGURE S2: Survival analysis of the PFS and OS of patients with *EGFR* 21L858R mutation with and without *BIM* deletion polymorphism, in the CTONG0901 cohort (A, C) and in the GLCI cohort (B, D). *BIM* del: *BIM* deletion; *BIM* wt: wild‐type *BIM*.

FIGURE S3: Genetic profiles of *EGFR*‐mutant patients with NGS results at baseline treated with first‐ and second‐generation EGFR‐TKIs. Only variations detected in at least three patients are shown.
