# Supplementary figures and images for: Concomitant genetic alterations having greater impact on the clinical benefit of EGFR‐TKIs in EGFR‐mutant advanced NSCLC than BIM deletion polymorphism
Source: Clin Transl Med. 2020 May 19;10(1):337–45. doi: 10.1002/ctm2.12 (PMC7240862; doi:10.1002/ctm2.12)

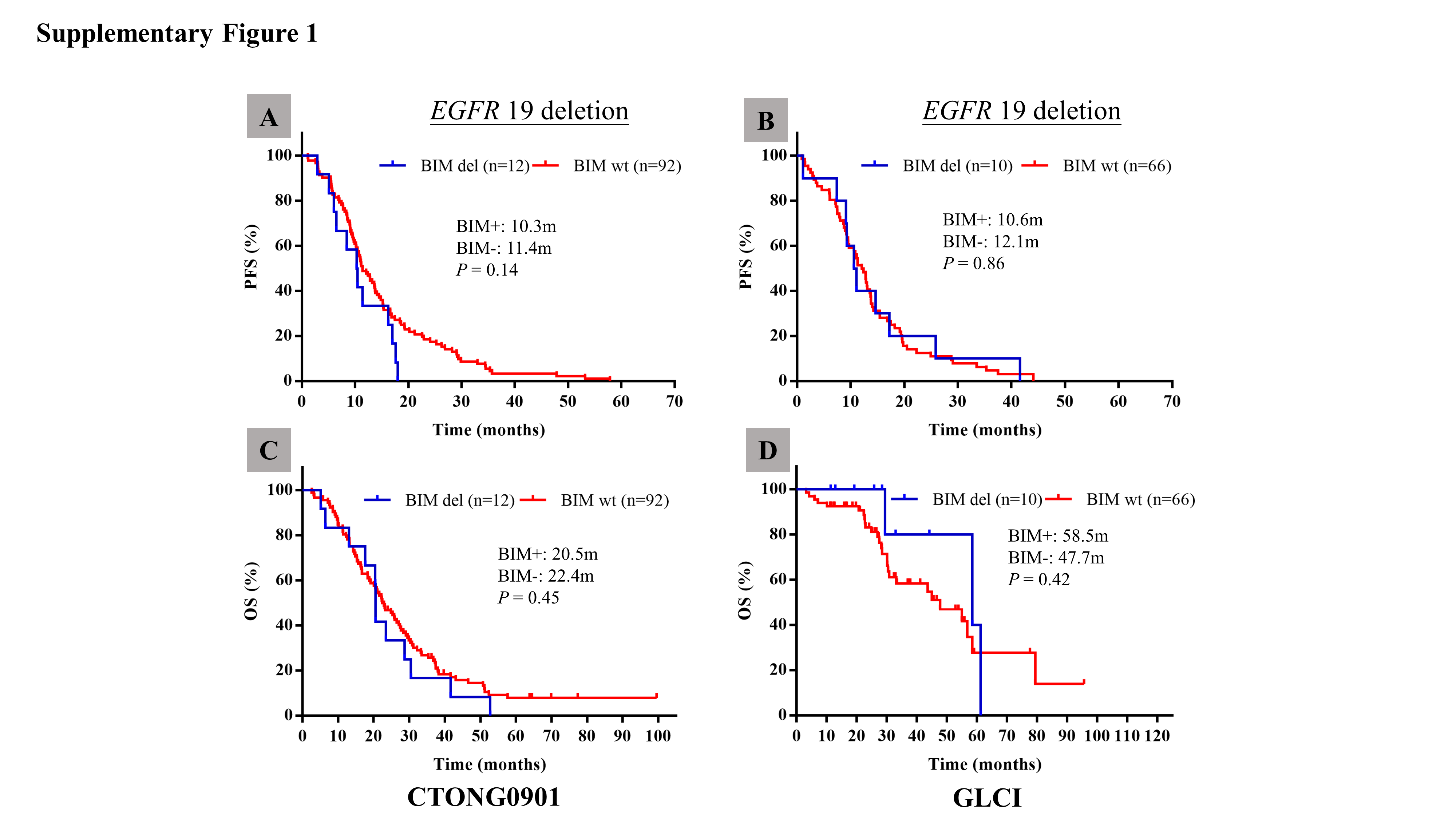

Supplement: Supplementary file 1 — FIGURE S1: Survival analysis of the PFS and OS of patients with EGFR 19 deletion with and without BIM deletion polymorphism, in the CTONG0901 cohort (A, C) and in the GLCI cohort (B, D). BIM del: BIM deletion; BIM wt: wild‐type BIM. [file CTM2-10-337-s001.TIF]

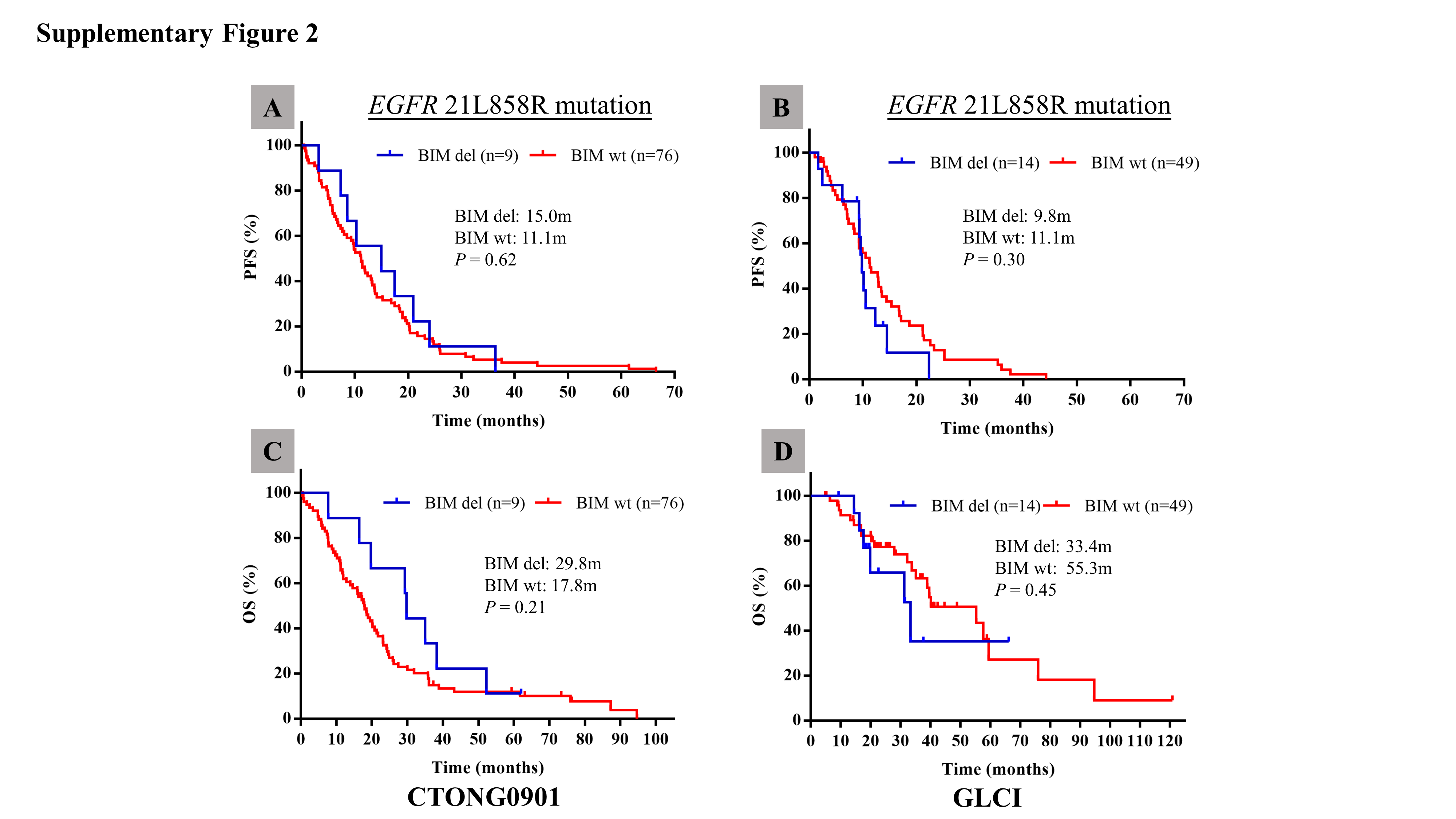

Supplement: Supplementary file 2 — FIGURE S2: Survival analysis of the PFS and OS of patients with EGFR 21L858R mutation with and without BIM deletion polymorphism, in the CTONG0901 cohort (A, C) and in the GLCI cohort (B, D). BIM del: BIM deletion; BIM wt: wild‐type BIM. [file CTM2-10-337-s002.TIF]

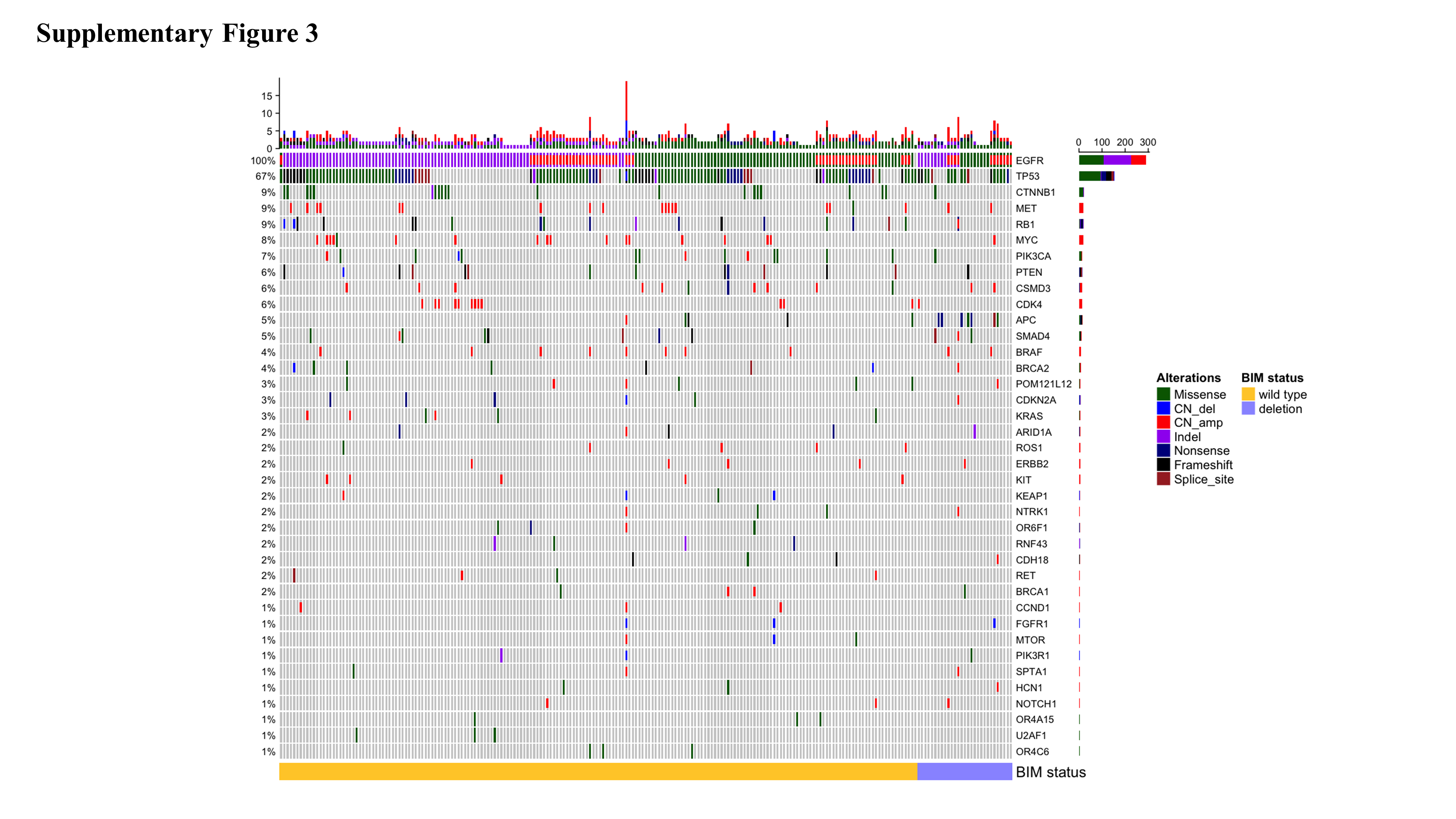

Supplement: Supplementary file 3 — FIGURE S3: Genetic profiles of EGFR‐mutant patients with NGS results at baseline treated with first‐ and second‐generation EGFR‐TKIs. Only variations detected in at least three patients are shown. [file CTM2-10-337-s003.TIF]
